# High-Precision Photonics-Assisted Two-Step Microwave Frequency Measurement Combining Time and Power Mapping Method

**DOI:** 10.3390/s24196415

**Published:** 2024-10-03

**Authors:** Zhangyi Yang, Zuoheng Liu, Yuqing Jiang, Hanbo Liu, Jiaqi Li, Wei Dong

**Affiliations:** 1 State Key Laboratory on Integrated Optoelectronics, College of Electronic Science and Engineering, Jilin University, Changchun 130012, China; yangzy21@mails.jlu.edu.cn (Z.Y.); zuoheng23@mails.jlu.edu.cn (Z.L.); jiangyq23@mails.jlu.edu.cn (Y.J.); hbliu23@mails.jlu.edu.cn (H.L.); 2 Shanghai Radio Equipment Research Institute, Minhang District, Shanghai 201109, China

**Keywords:** frequency measurement, frequency-to-time mapping, frequency-to-power mapping, microwave photonics

## Abstract

Photonics-assisted methods for microwave frequency measurement (MFM) show great potential for overcoming electronic bottlenecks and offer promising applications in radar and communication due to their wide bandwidth and immunity to electromagnetic interference. In common photonics-assisted MFM methods, the frequency-to-time mapping (FTTM) method has the capability to measure various types of signals, but with a trade-off between measurement error, measurement range, and real-time performance, while the frequency-to-power mapping (FTPM) method offers low measurement error but faces great difficulty in measuring signal types other than single-tone signals. In this paper, a two-step high-precision MFM method based on the combination of FTTM and FTPM is proposed, which balances real-time performance with measurement precision and resolution compared with other similar works based on the FTTM method. By utilizing high-speed optical sweeping and an optical filter based on stimulated Brillouin scattering (SBS), FTTM is accomplished, enabling the rough identification of multiple different signals. Next, based on the results from the previous step, more precise measurement results can be calculated from several additional sampling points according to the FTPM principle. The demonstration system can perform optical sweeping at a speed of 20 GHz/μs in the measurement range of 1–18 GHz, with a measurement error of less than 10 MHz and a frequency resolution of 40 MHz.

## 1. Introduction

Microwave frequency measurement (MFM) technology can provide frequency information for communication, radar, and electronic warfare systems and, therefore, often plays an important role in these fields [1]. With the increasing complexity of the electromagnetic environment, traditional electronic methods make it more difficult to meet the requirements of these systems in terms of working bandwidth, measuring speed, etc. In recent years, due to continuous advancements, such as a wide bandwidth and electromagnetic interference resistance, microwave photonics has received widespread attention, making it a key technology expected to overcome the electronic bottlenecks mentioned above. During this period, MFM technology based on microwave photonics has been widely studied, and the idea of mapping frequency onto various physical parameters that are easier to measure has led to the emergence of a large number of excellent solutions in this research field. These MFM methods can be categorized according to their measurement principles, including the frequency-to-power mapping (FTPM) method [2,3,4,5,6,7], frequency-to-time mapping (FTTM) method [8,9,10,11,12,13,14,15], and frequency-to-space mapping (FTSM) method [16,17]. Although the FTPM method offers high measurement precision and speed in measurements, it encounters challenges when applied to signals other than single-tone signals. In [2], a Brillouin phase–gain ratio was introduced to accomplish FTPM, and a measurement error below 5 MHz was achieved within two frequency bands of 0.12–0.17 and 10.12–10.17 GHz. The FTTM method correlates frequency with signals occurring at various time points, inherently possessing the capability for conducting multi-frequency measurements. The reported FTTM method can be further categorized based on its implementation approach, including the optical-domain Fourier scheme [8,9] and frequency-sweeping filtering scheme [10,11,12,13,14,15], both of which are capable of measuring various signal types. When compared with the optical-domain Fourier scheme, the implementation of a frequency-sweeping filtering scheme is relatively straightforward with higher performance and, thus, has garnered significant attention.

The typical frequency-sweeping filtering scheme consists of two primary elements: a chirp source and a narrowband filter. The signal under test (SUT) is modulated onto a specific optical sideband associated with time by a modulator. When this sideband passes through the passband of the filter, it maps different frequency information onto time-domain sequential electrical pulses with the help of a low-speed photodetector (PD); thus, the frequency of the SUT can be extracted by analyzing the time intervals between pulses and other relative parameter settings.

In some reported schemes [10,11,12,13,14], a known reference signal of fixed frequency is co-modulated with the SUT to create periodic reference pulses, serving as temporal markers to distinguish different sweeping cycles, which increases the complexity of the proposed schemes. In [10], periodic stimulated Brillouin scattering (SBS)-based FTTM was implemented by using a periodic nonlinear frequency-sweeping optical signal with a time-varying chirp rate, which enabled more accurate measurements in the band of interest and multiscale time–frequency analysis. It can be found that the full-width at half-maximum (FWHM) of pulses produced by the FTTM system has a direct impact on the MFM resolution and accuracy. Simultaneously, the bandwidth of the bandpass filter and the chirp rate of the sweep signal are significant factors affecting the pulse width.

Consequently, FTTM-based MFM methods exhibit a trade-off between real-time performance and measurement precision and resolution. High-speed frequency measurement and low measurement error and resolution cannot be simultaneously achieved, making the FTTM-based MFM methods mentioned above challenging to implement in systems with demanding requirements for both measurement speed and accuracy.

This paper aims to propose a new two-step high-precision MFM method that combines FTTM and FTPM. By utilizing high-speed optical-sweeping FTTM, supplemented by digital post-processing techniques, such as curve smoothing, pulse identification, and reference correction, a rapid coarse measurement of the SUT within a wide bandwidth is achieved. The measurement error at this stage is less than 50 MHz within the measurement range of 1–18 GHz when the chirp rate is no more than 20 GHz/μs. After this, an additional fine measurement stage based on the FTPM principle is performed to obtain a more accurate frequency of the SUT. Specifically, based on the coarse measurement result of the SUT, by taking advantage of the narrow bandwidth and Lorentz shape of the Brillouin gain spectrum (BGS), the precise frequency of the SUT is calculated using the FTPM method through different gains of a series of probe signals with sidebands located in the BGS. The experimental results show the measurement error over the measurement range of 1–18 GHz is further reduced to less than 10 MHz, and the measurement resolution is reduced to 40 MHz. In summary, the introduction of the fine measurement stage balances the real-time performance with measurement precision and resolution compared with other similar work based on the FTTM method.

The principle of the proposed scheme will be described in detail in Section 2. The details of the demonstration system and experimental results will be expressed in Section 3. There will be a discussion about the results and a comparison with other similar works in Section 4. Section 5 is the conclusion of this paper.

## 2. Principle of Operation

**Figure 1:**
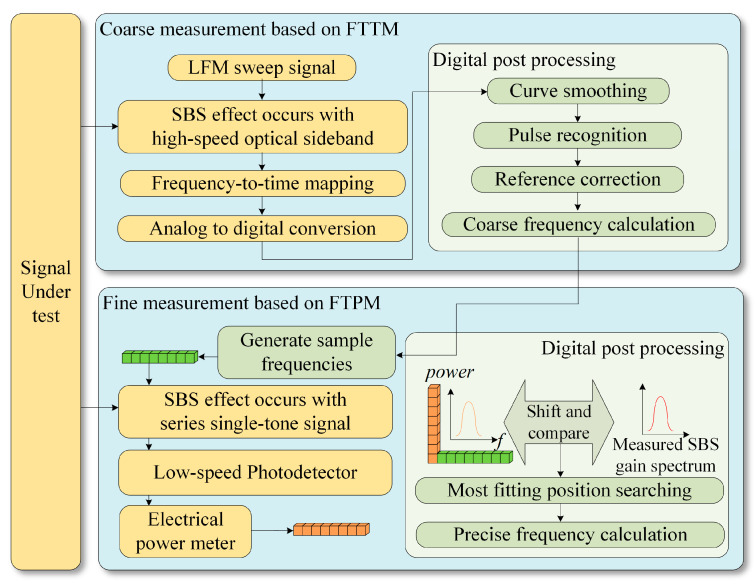
Flowchart of the measurement procedure of the proposed MFM system.

The measurement flowchart of the proposed MFM system is shown in Figure 1. The SUT needs two sequential steps to be accurately measured. The first step is coarse measurement based on FTTM. During this stage, the procedure maps frequency information onto low-speed temporal pulses across a broad band using an optical filter characterized by a narrow passband created through the SBS effect. When the instantaneous frequency of the linear frequency modulation (LFM) sweep signal is just within the BGS, which can be regarded as a narrowband optical filter, the optical power increases, thus generating a pulse. The electrical pulses are then converted into digital data through analog-to-digital conversion, and the coarse measurement of the SUT is achieved in digital post-processing, in which the steps of curve smoothing, pulse recognition, and reference correction are applied. By calculating the time difference between the pulses, the coarse frequency of the SUT can be obtained.

In the next stage, a sequence of single-tone frequencies is generated based on the coarse measurement result and then modulated as sidebands on the optical carrier. Depending on the position of these sidebands in the BGS, the gain obtained varies. These single-tone signals resemble a set of probes positioned in proximity to the BGS generated by the sidebands corresponding to the SUT. Consequently, the electrical power of the signal obtained after low-speed PD differs. Multiple sample points near the center frequency of the SBS-based filter can be obtained, and then the accurate frequency of the SUT can be determined using the FTPM method.

### 2.1. Coarse Measurement Stage Based on FTTM

**Figure 2:**
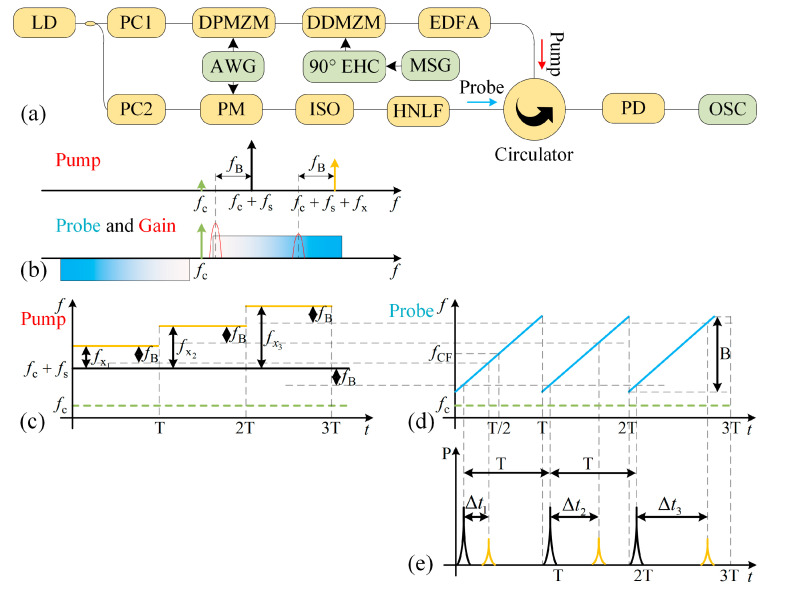
Details about the proposed scheme. (**a**) The diagram of the proposed MFM system. (**b**) The optical spectrum of the pump and probe light when the SUT is a single-tone signal. The pump wave (**c**), probe wave (**d**), and PD output (**e**) when the SUT is a step-swept signal.

Based on the measurement flowchart described above, a system shown in Figure 2a was designed to implement them. The optical carrier generated by a laser diode (LD) is split into upper and lower branches through an optical coupler. After this, the two branches pass through the polarization controllers (PCs), respectively, to make the modulator achieve the highest modulation efficiency.

In the upper branch, a drive signal $f_{s}$ generated by the arbitrary waveform generator (AWG) is modulated on the optical carrier $f_{c}$ using a dual–parallel Mach–Zehnder modulator (DPMZM), which is configured for carrier-suppressed single-sideband (CS-SSB) modulation by adjusting the bias voltages. The output of the DPMZM is then injected into the dual-drive Mach–Zehnder modulator (DDMZM) as the carrier, undergoing single-sideband (SSB) modulation through the SUT generated by the microwave signal generator (MSG) after a 90-degree electrical hybrid coupler (90° EHC). The output of the DDMZM is then amplified by an erbium-doped optical fiber amplifier (EDFA) and functions as the pump wave shown in Figure 2b. It is noted that the purpose of using DPMZM is to shift the optical carrier to an appropriate frequency in order to generate a reference time point in the output waveform of the system.

In the lower branch, the optical carrier is applied to a phase modulator (PM), which is driven by a chirp signal $f_{sweep}$ that has a center frequency of $f_{CF}$, a bandwidth of B, and a period of T in the coarse measurement stage or a sequence of single-tone frequencies in the next stage. It is noted that the signal here is generated by another channel of the AWG, which is different from the drive signal $f_{s}$. The output of the PM acts as the probe wave, which is sent to a coil of high non-linear fiber (HNLF) via an optical isolator (ISO). The pump wave from the upper branch enters the optical circulator and is counter-injected into the lower branch. It counter-propagates with the probe wave in the HNLF, leading to the excitation of the SBS effect. Then, the probe wave amplified by the BGS leaves the optical circulator and is detected by the low-speed photodetector (PD). The output of the PD is observed by an oscilloscope (OSC) and recorded as waveforms for further processing.

In the above process shown in Figure 2b, the optical carrier $f_{c}$ (green arrow) is shifted to $f_{c}+f_{s}$ (black arrow) as the frequency-shifted carrier through the CS-SSB modulation, which works as a fixed sideband of the pump to generate a pulse as a reference time point in the output of the PD. Subsequently, the sideband at a frequency of $f_{c}+f_{s}+f_{x}$ (orange arrow) is obtained through SSB modulation by the SUT with a frequency of $f_{x}$. When the chirp signal $f_{sweep}$ sweeps to $f_{s}+f_{x}-f_{B}$, the probe wave is amplified by the BGS centered at $f_{c}+f_{s}+f_{x}-f_{B}$, where $f_{B}$ is the Brillouin frequency shift.

As the SUT is loaded into the optical domain with SSB modulation, by adjusting the bias voltage of the DDMZM and the EDFA gain appropriately, it is achieved that only the frequency-shifted signal (black arrow) and the sidebands associated with the SUT (orange arrow) exceed the Brillouin threshold, and the power of the frequency-shifted signal has the maximum power in the pump wave. The pulses with a period of T and relatively large amplitude are called the reference pulses, and the others are called signal pulses. The reference pulses serve as the temporal reference, so it does not need to consider time synchronization issues between instruments, and the frequency of the SUT is mapped onto the time difference instead of the absolute time. It should be noted that $f_{s}-f_{B}$ should be slightly larger than $f_{CF}-B/2$ to ensure the reference pulse occurs in each period. The SUT should last at least one time period for the chirp signal to be detected, so the time resolution of the proposed system is T. To ensure the center frequency of the BGS is in the sweep range of the chirp signal, the measurement range of the proposed system is limited to the bandwidth B of the chirp signal.

When the SUT is a multi-tone signal, there is a signal pulse for each tone in a single period. When the SUT is a step-frequency signal, the time–frequency diagram of the pump wave and the probe wave is shown in Figure 2c,d, and the pulses from the PD are shown in Figure 2e, in which the grey dashed lines represent the correspondence between the pulses and the frequencies. As we see, the frequency $f_{C}+f_{S}$ in the pump generates the black reference pulses in Figure 2e, and the orange pulses correspond to the instantaneous frequency of the SUT. The time difference between the reference pulses and the signal pulses are $\Delta t_{1}$, $\Delta t_{2}$, and $\Delta t_{3}$, so the coarse measurement frequency can be calculated as:

(1) $$f_{xn}=\frac{B}{T}\cdot \Delta t_{n}=CR\cdot \Delta t_{n}$$

where CR is the chirp rate of the sweeping signal.

### 2.2. Fine Measurement Stage Based on FTPM

In the fine measurement stage, a series of single-tone signals is applied to the radio frequency (RF) port of the PM as a probe, and the output of the PD is recorded with the oscilloscope as waveforms, from which the power corresponding to a single-tone signal can then be extracted. Before the measurement, it is necessary to apply a series of single-tone signals $f_{ps}=[f_{ps1},f_{ps2},\ldots ,f_{psN}]$ with fixed frequency intervals $f_{i}$ using the method mentioned above without applying any SUT to obtain the shape of the BGS. The center frequency of $f_{ps}$ can be expressed as $f_{c}+f_{s}-f_{B}$, as shown in the red line area on the left in Figure 2b. The corresponding power $P_{ps}=[P_{ps1},P_{ps2},\ldots ,P_{psN}]$ is measured to record the shape of the BGS excited by the sideband with frequency $f_{c}+f_{s}$, where $\left(f_{psmax},P_{psmax}\right)$ is the peak. Based on the data of the measured BGS, it becomes feasible to precisely determine the location of the peak through the analysis of a limited number of sampling frequencies and their respective power levels.

After obtaining the coarse measurement result $f_{coarse}$ based on FTTM, the approximate center frequency of the BGS corresponding to the SUT can be expressed as $f_{c}+f_{s}+f_{coarse}-f_{B}$. Therefore, if a series of sampling point frequencies centered at $f_{s}+f_{coarse}-f_{B}$ with a fixed step is applied to the PM, some of these will fall at different positions of the BGS, and the electrical output of the PD will have different power levels.

First, using the method mentioned above, the corresponding power of the output signal can be obtained. Given that the average power of the PD output remains consistent when the probe sidebands fall outside the BGS, it becomes feasible to establish a suitable threshold for filtering out sampling frequencies inside the BGS, which is called valid samples. Normally, the maximum and minimum sampling point frequencies generated are not valid samples, so the threshold can be determined by the average of the two corresponding power levels. These valid sampling frequencies are labeled as $f_{p}=\left[f_{p1},f_{p2},\ldots ,f_{pM}\right]$, accompanied by their respective power levels denoted as $P_{p}=\left[P_{p1},P_{p2},\ldots ,P_{pM}\right]$, so the frequency difference between each valid sample frequency and the first valid sample frequency can be denoted as $\Delta f_{p}=\left[\Delta f_{p1},\Delta f_{p2},\ldots ,\Delta f_{pM}\right]=[f_{p1}-f_{p1},f_{p2}-f_{p1},\ldots ,f_{pM}-f_{p1}]$.

Second, the L-th point of $f_{ps}$ is aligned with the first point of $f_{p}$ in order, and the L2 norm of the corresponding power is calculated, which can be denoted as:

(2) $$P_{p}^{L}=\sum_{M}{\left(P_{pM}-P_{pLM}\right)}^{2}$$

in which $P_{pLM}$ represents the corresponding power in $P_{ps}$ when the frequency is $f_{sL}+\Delta f_{pM}$. If the SUT contains a dual-tone signal with a small frequency difference, $P_{p}$ contains more points than $P_{ps}$, which will cause the above process to fail to execute correctly. The $P_{p}$ can be divided into two parts according to the interval between the two peaks in it and then processed separately.

Finally, the fine measurement result can be calculated as:

(3) $$f_{fine}=f_{p1}+\left(f_{psmax}-f_{ps1}\right)-L_{min}\cdot f_{i}$$

where $L_{min}$ represents the parameter value of L when $P_{p}^{L}$ reaches its minimum value.

## 3. Experimental Results

A proof-of-concept system was constructed to validate the proposed system. The laser emitted by the LD (Santec, TSL-550) centered at 1549.988 nm with a power of 10 dBm is split into two branches by an optical coupler with power ratio of 8:2. In the upper branch, the laser is injected into the DPMZM via PC 1, which is CS-SSB modulated with a pair of in-phase quadrature (IQ) signals generated by the AWG (Keysight, M8195A). The output of the DPMZM is then injected into the DDMZM (Fujitsu, FTM7937EZ200) biased at the quadrature point as the frequency-shifted carrier, undergoing SSB modulation with the SUT generated by the MSG (Keysight, E8257D) after the 90° EHC. The output of DDMZM is then amplified by EDFA and functions as the pump wave. The carrier in the lower branch is injected into a PM via PC 2 and modulated by a chirp or single-tone signal generated by another channel of the AWG. The output of the PM serves as probe wave via ISO and enters a 1 km long HNLF, with the SBS interaction effect with the pump wave propagating in the reverse direction through the optical circulator. The ISO here can block the pump wave to avoid damaging the device. Then, the probe wave after the SBS interaction enters the low-speed PD, and the electrical output is observed and recorded by the OSC (Keysight, MSOV254A).

### 3.1. Coarse Measurement Result

**Figure 3:**
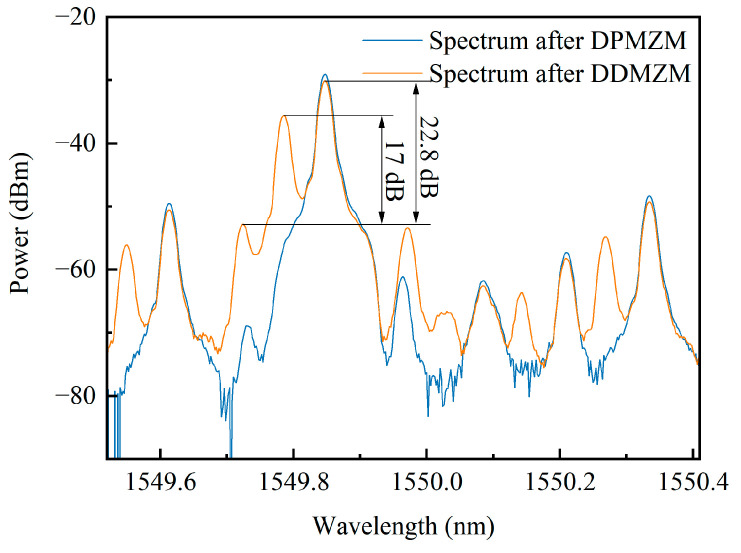
The spectra of DPMZM and DDMZM outputs.

During the experiment, a pair of IQ signals from the AWG with the frequency $f_{s}=15\;GHz$ is used as the drive signal of the DPMZM to achieve the frequency shifting of the optical carrier, and an 8 dBm microwave signal from the MSG with the frequency $f_{x}=10\;GHz$ serves as the SUT. The optical spectrum of the DPMZM and DDMZM outputs are shown as the blue and red lines in Figure 3, respectively. The frequency-shifted carrier still maintains the highest sideband, which results in the reference pulses having the maximum amplitude, thus distinguishing signal pulses from reference pulses based on this criterion. Among the remaining sidebands, although the positive and negative third-order sidebands output by the DPMZM have relatively high power, the potential SBS gain or loss spectrum does not fall within the sweeping range of the probe wave and, therefore, no interference with the system occurs. In the spectrum from the DDMZM, the useful sideband surpasses the highest spurious sidebands by 17 dB, so it can be achieved by adjusting the EDFA gain to ensure that only the frequency-shifted carrier and the corresponding sideband of the SUT exceed the SBS threshold, thereby avoiding interference pulses.

A LFM signal from another channel of the AWG, characterized by a 4 μs period, a central frequency of 15.6 GHz, and a 20 GHz bandwidth, serves as the driving signal for the PM to generate the probe wave. Hence, the chirp rate of the probe wave is 5 GHz/μs. Due to the SBS frequency shift being 9.3880 GHz in our experimental environment, the above experimental parameter settings can result in the center frequency of the BGS generated by the frequency-shifted optical carrier being slightly higher than the starting frequency of the probe sweeping wave.

**Figure 4:**
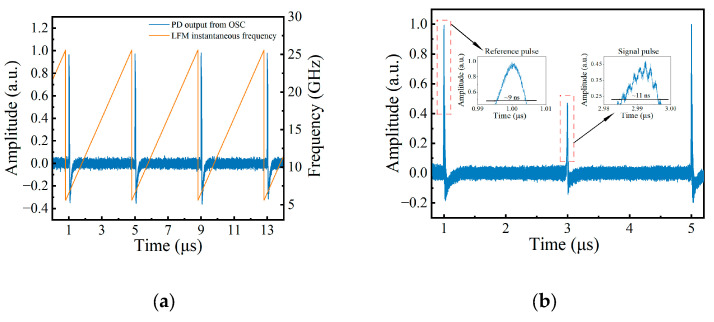
The output of PD when performing coarse measurement stage. (**a**) The output pulses and corresponding LFM frequency without any SUT; (**b**) the output pluses when the SUT is 10 GHz.

When the MSG output is turned off, that is, without applying any SUT on the DDMZM, the oscilloscope can still detect the periodic reference pulses illustrated in Figure 4a, in which the orange line illustrates the alignment between the pulse and LFM instantaneous frequency. The pulse amplitudes exhibit stability and are distinguishable. The temporal gap between any consecutive pair of pulses is uniform, aligning with the period of the sweeping probe signal.

**Figure 5:**
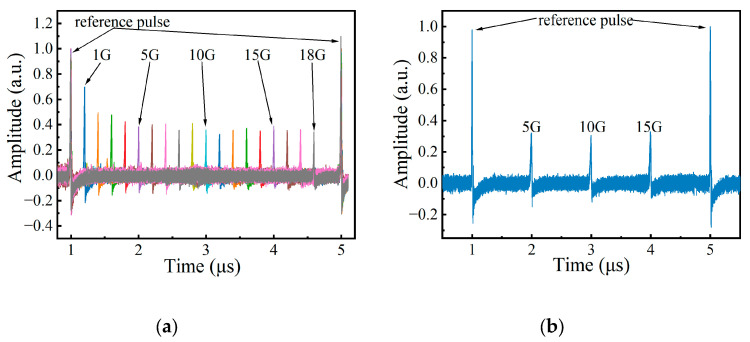
Waveforms from OSC for different types of signals as SUT. (**a**) The output pulses when the SUT is from 1 to 18 GHz with a frequency step of 1 GHz; (**b**) the output pulses when the SUT is a multi-tone signal.

When the MSG output is turned on, the signal output from the oscilloscope is shown in Figure 4b. If choosing the peak of the pulse as the reference point for calculating the time difference, the time difference is 2.0046 μs, and the SUT is calculated as 10.023 GHz. Figure 5a illustrates the output waveforms when the frequency of SUT is 1 GHz, 2 GHz, 3 GHz, …, and 18 GHz, respectively. The figure was created by overlaying the 18 curves corresponding to 1 GHz, 2 GHz, 3 GHz, …, 18 GHz on the same axis. The reference pulses of each curve in the figure are aligned to the same position (1 μs and 5 μs). It can be found that the signal pulses and reference pulses in Figure 5a can be clearly distinguished from the noise, proving that the proposed system has a measurement range of 1–18 GHz. The ability of the proposed system to measure multi-tone signals was also tested. A multi-tone signal including 5 GHz, 10 GHz, and 15 GHz generated by AWG was used as the SUT, and the signal output from the oscilloscope is shown in Figure 5b. The frequencies of the multi-tone SUT can be calculated as 4.9990 GHz, 9.9970 GHz, and 15.0045 GHz using Equation (1).

**Figure 6:**
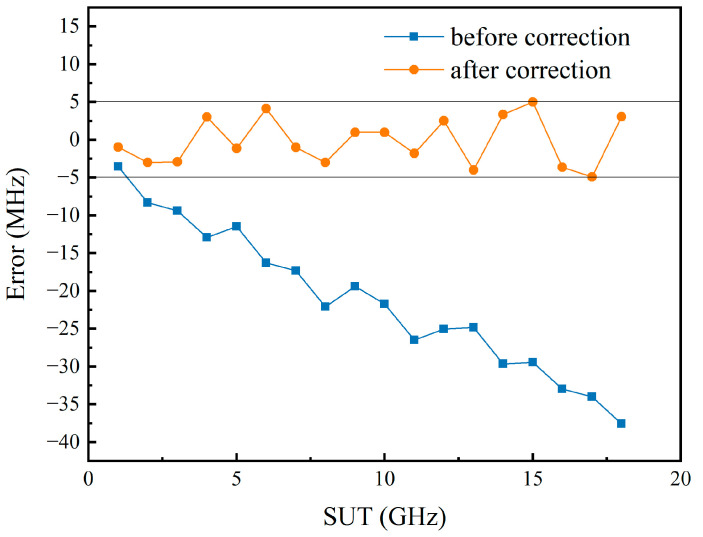
The measurement error of the SUT before and after post-processing when the chirp rate of the sweeping signal is 5 GHz/μs.

In previous calculations, the moment when the peak of the pulse appears was used to calculate the time difference. However, from Figure 4b, it can be seen that the pulse width is approximately 10 ns, and there are some spikes in the pulses, making it difficult to determine the moment of pulse occurrence. Additionally, the signal pulse exhibits an asymmetrical shape, indicating that it does not reach its maximum value at its center. Obviously, this method is not precise enough, these factors are sufficient to have a noticeable impact on the measurement results of the SUT. Moreover, due to the imperfections of the signal source used, there is often a certain error between the actual chirp rate of the swept signal and the set value. This leads to an increase in the measurement error as the frequency of the SUT increases. Therefore, the Savitzky–Golay [18] algorithm is applied to the acquired signal for curve smoothing. Since the filtered signal is very smooth and the pulse width is stable, the pulse recognition can be accomplished by using the findpeaks function of MATLAB R2022b. Then, signals with known frequencies are used as the SUT to obtain the Δt of known frequencies to perform reference correction. The actual chirp rate of the sweeping signal is calculated using Equation (1). The blue line and orange line in Figure 6 represent the measurement errors of the SUT with and without the post-processing, respectively.

**Figure 7:**
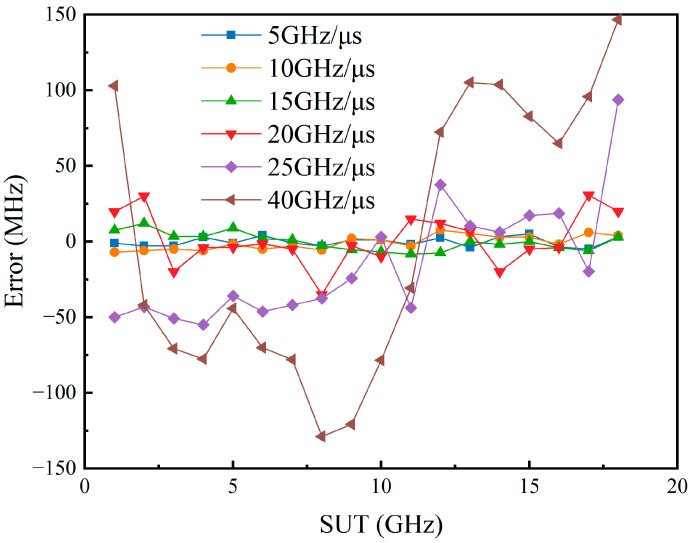
The measurement error of the SUT after correction when the chirp rate of the probe wave is 5 GHz/μs, 10 GHz/μs, 15 GHz/μs, 20 GHz/μs, 25 Hz/μs, and 40 GHz/μs.

As the DPMZM is utilized to shift the carrier in the frequency domain, the limitations on the measurement range imposed by the SBS frequency shift are removed. Consequently, a broader bandwidth of the chirp signal indicates an expanded measurement range. Considering the real-time capabilities of the system and the potential for optical chirp sideband generation, the measurement error of the SUT after correction was tested at chirp rates of 5 GHz/μs, 10 GHz/μs, 15 GHz/μs, 20 GHz/μs, 25 GHz/μs, and 40 GHz/μs. This was accomplished by adjusting the period while maintaining the other parameters of the chirp signal unchanged, and the results are shown in Figure 7.

When the chirp rate is less than 15 GHz/μs, curve smoothing and reference correction can still yield relatively accurate results. However, since the pulse width is mainly affected by the chirp rate at this time [14], the pulse width increases as the chirp rate increases. These factors contribute to the rise in measurement errors of the SUT.

### 3.2. Fine Measurement Result

**Figure 8:**
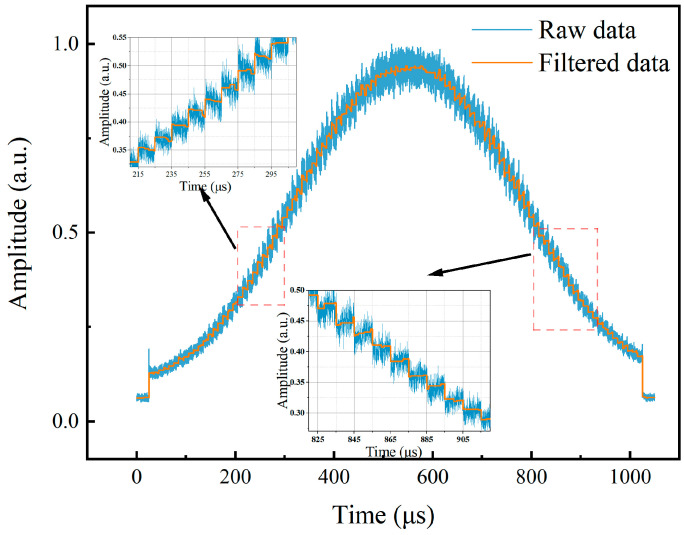
The shape of the BGS.

According to the fine measurement principle, in the absence of the SUT, the PM’s drive signal varies within the range of 5.5870 GHz to 5.6370 GHz with a 0.5 MHz step to obtain the shape of the BGS. In the experiment, a step-swept signal was used as the drive signal of PM to complete this step. The duration of the signal was 1050 μs, with 25 μs of silence before and after as indicators for different periods. Therefore, the duration of each frequency point was 10 μs. The signal received by the oscilloscope is shown as the blue line in Figure 8. The orange line is the result of the signal after sliding filtering, from which the power information can be extracted and prepared for the fine measurement stage. It can be observed that both the ascending and descending parts of the BGS have adequate slopes to distinguish the frequency changes in the probe wave as low as 0.5 MHz.

**Figure 9:**
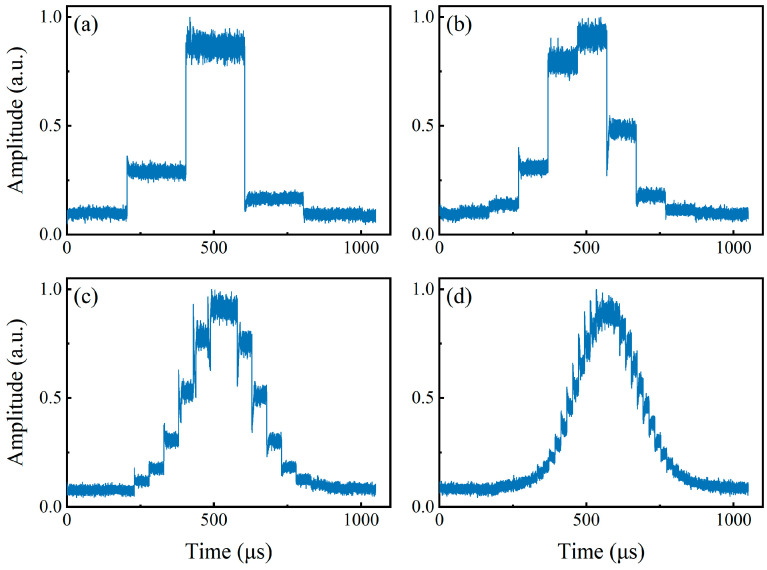
The output waveforms when the frequency interval of the step-swept signal is (**a**) 20 MHz, (**b**) 10 MHz, (**c**) 5 MHz, and (**d**) 2 MHz.

Next, based on the coarse measurement results, step-swept signals with different frequency intervals were generated by the AWG to implement the process of modulating a series of single-tone signals up to the PM. The output signals acquired are shown in Figure 9. This sequence of different frequency points in the step-swept signal, like probes, explores the position of the BGS excited by the corresponding sidebands of the SUT. From the output signal, a sequence of power corresponding to different probe frequencies can be extracted, and the fine measurement result can be calculated using the method mentioned in the principle part.

**Figure 10:**
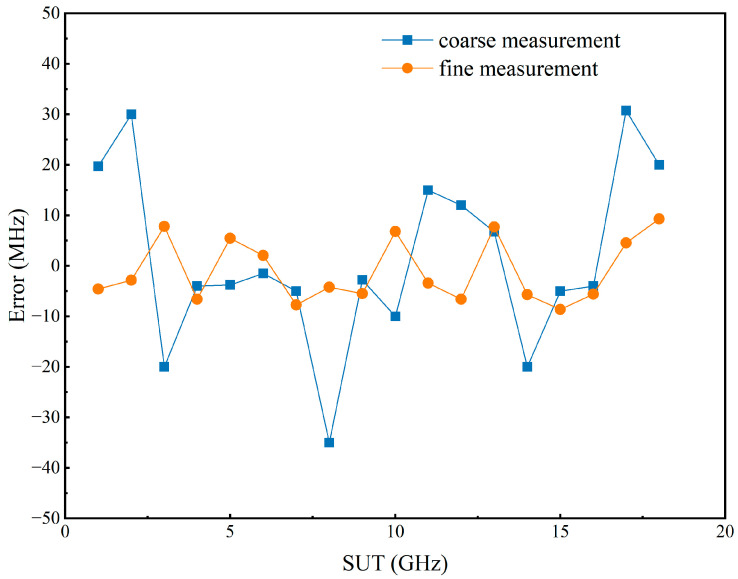
The coarse and fine measurement results when the chirp rate is 20 GHz/μs.

In fact, as long as the coarse measurement results do not deviate too much to the extent that the single-tone frequencies generated in the second stage do not fall within the BGS excited by the corresponding sidebands of the SUT, there is no significant impact on the fine measurement result. In the experiment, the coarse measurement result of the single-tone SUT at a chirp rate of 20 GHz/μs was selected for fine measurement, the frequency interval of the second stage was set to 10 MHz, and the results are shown in Figure 10. It is demonstrated that the fine measurement step can further reduce the system measurement error from about 40 MHz to a level below 10 MHz.

**Figure 11:**
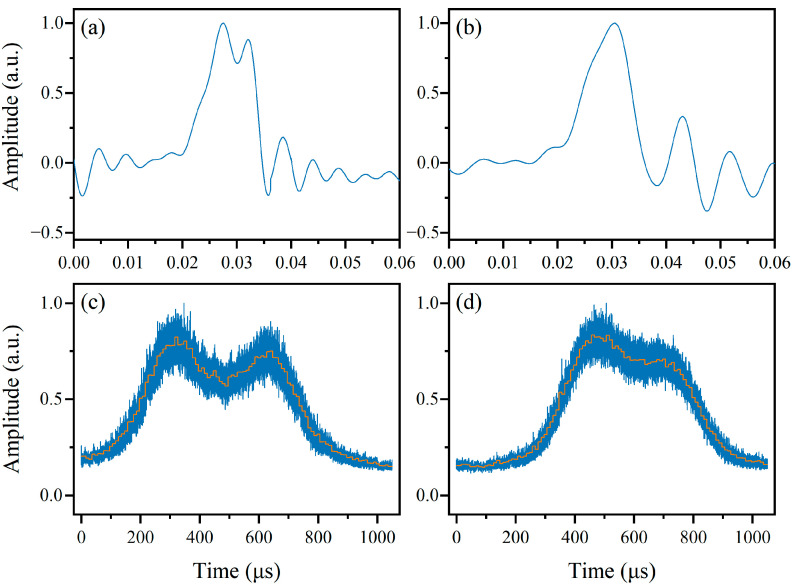
Comparison of resolution between the two measurement stages. The frequency differences in the dual-tone SUT are (**a**) 100 MHz; (**b**) 80 MHz; (**c**) 40 MHz; and (**d**) 35 MHz.

At last, the frequency resolution of the system was tested. The resolution of the coarse measurement was first tested as a baseline for comparison. Two dual-tone signals with frequency differences of 100 MHz and 80 MHz were tested when the chirp rate of the sweeping signal was 20 GHz/μs, and the waveforms from the OSC after curve smoothing are shown in Figure 11a,b, respectively.

Although the curve smoothing effectively removes the spikes in the raw signal, the dual-tone SUT with similar frequencies is still difficult to distinguish. Then, in the fine measurement stage, a step-swept signal with 100 frequency points and a period of 1050 μs is used as the drive signal of the PM to measure two dual-tone signals with frequency differences of 40 MHz and 35 MHz, and the results are shown in Figure 11c,d. It is demonstrated that the fine measurement stage can significantly improve the frequency resolution of the system when the chirp rate of the sweeping signal is relatively high (20 GHz/μs), from about 100 MHz to 40 MHz.

**Figure 12:**
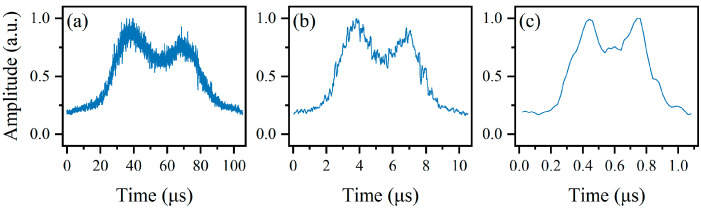
The waveforms from OSC when the durations of a single frequency point are (**a**) 1 μs, (**b**) 0.1 μs, and (**c**) 0.01 μs.

Furthermore, another experiment was conducted to test the real-time performance of the fine measurement stage. When the duration of a single frequency point in the step-swept signal is further reduced to 1 μs, 0.1 μs, and 0.01 μs, and the frequency difference in the dual-tone SUT is kept at 40 MHz, the results are shown in Figure 12, from which it can be seen that the duration of single frequency in the swept signal does not affect the amplitude of the PD output much, which means a faster fine measurement stage can be achieved.

## 4. Discussion

In practical applications, the SUT cannot be a simple single-tone or multi-tone signal; it contains complex frequency components and a huge amount of information. A multi-tone signal can also be considered as being composed of multiple single-tone signals, so each component can be processed in a manner similar to that of a single-tone signal one by one. Typically, a specific application always works on a fixed frequency band, so we can use the coarse measurement result to monitor electromagnetic signals in space and only perform the fine measurement stage for signals that interest us.

From the structure of the demonstration system, the proposed system does not require a feedback structure (which is difficult to debug), nor require customized devices, such as a high-Q resonator. By simply adding an FTPM stage to the basis of the FTTM, a balance between the real-time performance with the measurement error and resolution can be achieved.

In summary, this paper proposes a two-step high-precision MFM method based on the combination of FTTM and FTPM, which has a measurement range of 1–18 GHz with a multi-tone measurement ability. Compared with the reported photonics-assisted microwave frequency measurement schemes, the proposed scheme combines the advantages of FTTM and FTPM and has the characteristics of a fast frequency-scanning speed and low measurement error. It overcomes the shortage to measure multi-tone signals of the FTPM-based scheme and balances the real-time performance with measurement precision and resolution compared with other FTTM-based similar works. A detailed comparison with other similar works is shown below in Table 1.

## 5. Conclusions

A two-step high-precision MFM method based on the combination of FTTM and FTPM is proposed in this paper. The proposed system can achieve multi-tone signal measurements within a wide bandwidth range of 1–18 GHz, with a measurement accuracy better than 10 MHz and a frequency resolution of 40 MHz when the chirp rate is 20 GHz/μs. More importantly, the fine measurement stage, which greatly improves the system measurement accuracy and frequency resolution, does not have a long time consumption or high accuracy requirements for the coarse measurement results. Therefore, by combining the FTTM and the FTPM, the contradiction between the real-time performance, measurement accuracy, and resolution in the existing FTTM-based photonic-assisted MFM schemes is resolved.

## Figures and Tables

**Table 1:** Comparison of different frequency-sweeping filter MFM methods.

Ref.	Technique	Accuracy (MHz)	Resolution (MHz)	Sweep Rate (GHz/μs)	Frequency Range (GHz)
[9]	SBS-based channelized FTTM	Not tested	35	8	0–12
[11]	Two-step MFM	100	700	20	0–12
		30	70	2	Not tested
		20	70	0.2	Not tested
[12]	SBS-based FTTM	1	40	$5\times 10^{-5}$	6–18
[13]	Optimized FTTM	7.53	40	$2\times 10^{-4}$	16–26
[14]	SBS-based FTTM	4	105	4	0–4
[15]	Equivalent frequency sampling	6.9	46	0.75	30–33
[19]	High Q-factor microdisk	10	Not tested	$2\times 10^{-4}$	14.25–17.25
[20]	Integrated scanning filter	237.3	375	$2.9\times 10^{-3}$	1–30
[21]	OEO and microdisk resonator	100	200	2.74	0–20
[22]	FDML OEO	60	60	0.18	0–15
This work	FTTM and FTPM	10	40	20	1–18

## Data Availability

The data presented in this study are available upon request from the corresponding author.
